# Multiple brain metastases - current management and perspectives for treatment with electrochemotherapy

**DOI:** 10.2478/v10019-012-0042-y

**Published:** 2012-11-09

**Authors:** Mette Linnert, Helle K. Iversen, Julie Gehl

**Affiliations:** 1 Center for Experimental Drug and Gene Electrotransfer (C*EDGE), Department of Oncology, Copenhagen University Hospital Herlev, Herlev, Denmark; 2 Department of Neurology, Copenhagen University Hospital Glostrup, Glostrup, Denmark

**Keywords:** brain metastases, electroporation, bleomycin, electrochemotherapy, electrode device, blood-brain barrier

## Abstract

**Background:**

Due to the advanced oncological treatments of cancer, an overall increase in cancer incidence, and better diagnostic tools, the incidence of brain metastases is on the rise. This review addresses the current treatment options for patients with multiple brain metastases, presenting electrochemotherapy (ECT) as one of the new experimental treatments for this group of patients.

**Conclusions:**

Neurosurgery, stereotactic surgery, and whole-brain radiotherapy are the evidence-based treatments that can be applied for patients with multiple brain metastases. Treatment with chemotherapy and molecularly targeted agents may also be warranted. Several experimental treatments are emerging, one of which is ECT, an effective cancer treatment comprising electric pulses given by electrodes in the tumor tissue, causing electroporation of the cell membrane, and thereby augmenting uptake and the cytotoxicity of the chemotherapeutic drug bleomycin by 300 times. Preclinical data are promising and the first patient has been treated in an ongoing clinical trial for patients with brain metastases. Perspectives for ECT in the brain include treatment of primary and secondary brain tumors as well as soft tissue metastases elsewhere.

## Introduction

An increasing number of cancer patients develop brain metastases. Several factors may be responsible, including advancing age of the population, causing an overall increase in cancer incidence. Additionally, improved treatment of systemic cancer disease is leading to longer survival and thereby increasing the possibility of patients living long enough to develop brain metastases. The true incidence of brain metastases in the cancer patient population is difficult to estimate due to poor registration of this particular affliction in most countries. However, a rather precise number for the incidence of brain metastases in the Metropolitan Detroit area between 1973 and 2001 was 9.6%, with the highest incidence in lung cancer patients (19.9%) and lowest for patients with colorectal cancer (1.8%).^1^ In Sweden, the number of admissions due to brain metastases doubled from 1987 to 2006, with the largest increase in patients with lung and breast cancer.^2^

**FIGURE 1.**
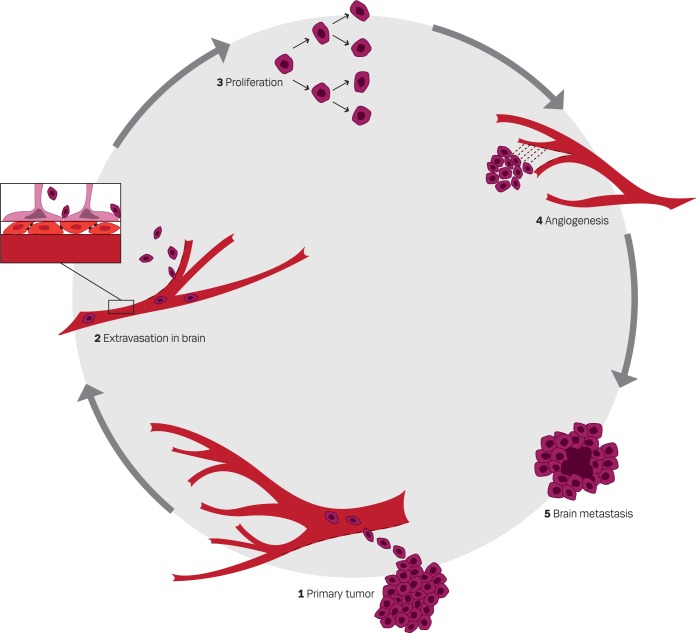
Pathogenesis of brain metastases.

Brain metastases are developed through a long line of steps: tumor cells from the primary tumor enter the blood stream and reach the brain, attach to the endothelial cells, and extravasate into the brain parenchyma to proliferate and induce angiogenesis, see Figure 1.^3^ Each step along this path to the development of a brain metastasis may represent targets for treatments at hand or in the future.

With modern diagnostic tools such as magnetic resonance imaging (MRI), patients can be diagnosed earlier in the course of the disease. Furthermore, using MRI as the diagnostic tool reveals that 80% of the patients have more than one brain metastasis and around 50% have three or more brain metastases.^4^ Schellinger *et al*. found that CT scan failed to demonstrate multiple brain metastases in 31% of patients screened with both CT and MRI scans.^5^

The time of occurrence of brain metastases varies greatly within different cancer types, often occurring late with breast cancer and colorectal cancer, while lung cancer patients frequently develop brain metastases early in the disease, maybe even at the time of diagnosis.^6^,^7^ Common symptoms of brain metastases are headache, paresis, and psychological changes, which are seen in about half the patients.^8^ Many patients also suffer from symptoms of elevated intracranial pressure such as nausea, vomiting, and headache. Up to 20% of the patients have trouble walking and/or suffer from seizures.^8^

So far, the primary goal of most treatments of brain metastases is palliation and focuses on improvement of symptoms and the patient’s quality of life. It has been shown that a reduction of tumor volume after whole brain radiation therapy is correlated to both improvement of the neurocognitive function and survival. Neurocognitive function is also closely related to the patient’s ability to cope with activities of daily living (ADL), which again is important for the quality of life.^9^ Therefore, reducing tumor volume is the goal of most treatments for brain metastases.

Novel molecularly targeted agents have shown activity against brain metastases, but so far nothing has revolutionized the treatment of brain metastases. Another upcoming treatment option could be electrochemotherapy (ECT), a treatment comprising of chemotherapy facilitated into the cancer cells by electric pulses that causes transient permeabilization of the cell membrane *e.g*. electroporation. The electric pulses are delivered by electrodes penetrating and covering the brain metastasis, causing electroporation of the cancer cells, and leading to a highly augmented uptake of the chemotherapeutic drug. The chemotherapeutic drug mostly used is bleomycin, which can be administered either intravenously or injected directly into the tumor. ECT has been thoroughly clinically tested as treatment for cutaneous tumors, is preclinically tested in a rat tumor model with promising results^10^, and a first-in-man clinical trial is now open for patients with brain metastases at our center (ClinicalTrials.gov, number NTC 01322100).

### The role of surgery

Surgery can be an excellent treatment for patients with single or few brain metastases, but is rarely an option for patients with multiple brain metastases. The key parameters used to decide whether surgery may be an option are size, number, and localization of the brain metastases, and is ultimately evaluated on a case by case basis by a neurosurgeon.^11^ For instance, a large, surgically accessible metastasis greater than 3 cm and with complicating mass effect could be considered for surgical treatment, and need for a confirmation of a diagnosis can be an important pro for surgery.^11^ Surgery seems to be equally as effective as stereotactic radiosurgery for the management of suitable solid metastases less than 3 cm in diameter, however, this conclusion may be subject to selection bias, as patients selected for surgery may have a better prognosis to begin with.^12^

### The role of radiotherapy

Radiotherapy can be applied locally, as Stereotactic Radiation Surgery (SRS) to a limited number of brain metastases, or as the traditional whole brain radiation therapy (WBRT), and is sometimes combined with each other or with surgery. The combined approach is mostly used for patients initially presenting with multiple brain metastases treated with WBRT and relapsing with a single or a few brain metastases suitable for SRS. SRS treatment up front can also be followed by WBRT. Prophylactic whole brain radiation therapy is routinely applied to patients with small-cell lung cancer and limited disease, improving patient survival.^13^

SRS may be regarded as an option for patients with fewer than 3–4 brain metastases of less than 3–4 cm in diameter, and compared to surgery more variable locations may be treatable.^14^ The median survival after SRS is better than for WBRT, and this is probably due to the patients being sorted using the known prognostic factors of few metastases and better performance status.

WBRT alone is most often the standard of care for patients with multiple brain metastases, leading to improvement of symptoms in about half the patients and a median survival of 4–6 months.^15^ Approximately 50% of patients in RTOG (Radio Therapy Oncology Group) trials have disease control after 6 months with initial response rates of around 60%.^4^ Kalkanis *et al*. found in a review of 4 studies that if surgery is combined with WBRT, both tumor control at the original site and distant control in the brain is improved, but not the overall survival.^16^ When avoiding treatment of the whole brain, the lack of distant control in the brain can be managed by frequent imaging surveillance, enabling salvage therapy in case of recurrence or appearance of new brain metastases.^12^ Chang *et al*. found most patients with distant recurrences were clinically asymptomatic (18 of 21) and only detected on MRI, concluding that local treatments, such as SRS should be followed by close imaging monitoring afterwards.^14^ Conversely, Patchell *et al*. finds that WBRT should be given up front after localized treatments, because of the generally low toxicity of WBRT, and to spare the patient of neurologic deterioration that may be irreversible when progression occurs.^17^

More than half the patients who are treated for brain metastases will in their lifetime eventually present with progressive disease in the brain, and more and more often as the only site of progression.^18^ Patients present with multiple symptoms such as headache, motor deficit, impaired mentation and seizures, where headache being the most common presenting symptom at debut of brain metastases, while motor deficit is the most frequent symptom at recurrence.^19^ At recurrence the current strategy is combining available evidence-based treatments, such as WBRT and SRS or even repeating WBRT. Event though the role of re-irradiation of the whole brain for recurrent/progressive brain metastases is controversial, mainly due to the data on the subject being retrospective, published over a large number of years while the oncological treatments have evolved somewhat. A list of these studies is shown in Table 1.

The studies on re-irradiation of the whole brain may therefore have underestimated the treatment toxicity and treated different types of patients, with respect to disease status and performance status, which is known to influence patient survival.

### The role of chemotherapy

A major concern regarding chemotherapy as a treatment for brain metastases is whether or not the antineoplastic drug crosses the blood-brain barrier (BBB). Another issue to consider is whether the patients are chemotherapy-naïve or have received several lines of chemotherapy prior to the development of brain metastases, increasing the possibility of tumor resistance.^20^ A few cancers are responsive to treatment with chemotherapy in the brain, such as malignant melanoma, lymphoma and small cell lung cancer, but for most solid tumors, the role of chemotherapy for brain metastases remains undefined.

One chemotherapeutic agent known for years and able to penetrate the BBB in therapeutic concentrations is the oral alkylating drug temozolomide (TMZ). TMZ has demonstrated activity against both primary and secondary brain tumors, especially in patients with malignant melanoma brain metastases.^21^,^22^ Agarwala *et al*. tested the efficacy and toxicity of temozolomide on 151 radiotherapy-naïve patients with malignant melanoma, showing one patient with complete response (CR), 8 patients (5%) with partial response (PR), and 40 patients (26%) with stable disease in the brain.^21^ Significant anti-tumor effects of TMZ have also been shown in a group of patients with mixed diagnoses, when treated with TMZ plus WBRT compared to WBRT alone, although an overall survival benefit was never found.^23^–^25^ The lack of ability to show a survival benefit was hypothesized by Antonadou *et al.* to be due to the fact that in their phase-II trial of 45 evaluable patients, the majority of patients in both groups progressed and died of disease progression at the primary site or developed other systemic metastases.^25^ In a small study of 24 patients with breast cancer brain metastases, an objective response rate of 18% was observed with the treatment with TMZ and capecitabine, which is a promising result that needs further confirmation in larger trials.^26^

Other chemotherapeutic drugs have been tested, and one promising clinical trial is Newton *et al.*’s treatment with intra-arterial carboplatin and etoposide in patients with brain metastases from various primary cancer diagnoses, producing an objective response rate of 38% in 24 patients with tolerable toxicity, warranting further studies.^27^ Kiewe *et al.*’s treatment with topotecan and ifosfamide in 12 patients with brain metastases resulted in 1 patient with PR and 3 patients with stable disease (SD), but at a price of considerable hematotoxicity, discouraging further investigations into this treatment.^28^

### The role of molecularly targeted agents

Molecularly targeted agents are now available in the treatment of brain metastases, such as the oral tyrosine kinase inhibitor (TKI) lapatinib for HER-2-expressing breast cancer patients.^29^ These patients have a reported high risk of brain metastases, which may be due to the poor penetration through the BBB of the otherwise effective HER-2 targeting drug trastuzumab, in addition to the possibility that HER-2 –expressing breast cancer may be more biologically aggressive.^29^

Lapatinib is acting by binding the inactive form of the erbB2 and the epidermal growth factor receptor (EGFR), promoting cell growth inhibition and inducing apoptosis. The small molecule does penetrate the BBB and has shown efficacy in the brain in clinical trials, especially when combined with the chemotherapeutic drug capecitabine, and is now routinely used in the clinic.^30^,^31^

Sunitinib is another small molecule TKI, acting on vascular endothelial growth factor receptors (VEGFR) and platelet-derived growth factor receptors (PDGFR) among others with a direct anti-tumor and anti-angiogenic activity. The drug is playing a major role in the treatment of patients with renal cell carcinoma (RCC) in general, and it is also in this patient group the best results are found in the treatment of brain metastases. A study of 321 patients with brain metastases from RCC found an overall response rate of 12% and a clinical benefit rate of 64%.^32^ Sunitinib has also been investigated in heavily pretreated breast cancer patients by Burstein *et al.*, resulting in 7 out of 64 patients having a partial response (PR), demonstrating activity and warranting further investigation.^33^ Unfortunately, a phase II study of 64 patients with non-small cell lung cancer showed only marginal anti-tumor activity.^34^

The EGFR tyrosine kinase inhibitors, gefitinib and erlotinib, are considered active agents in a subset of patients with non small-cell lung cancer harboring EGFR-mutations and seems to be effective in the treatment of brain metastases.^35^,^36^

Bevacizumab is a monoclonal antibody that binds to and inhibits the activity of VEGF and possibly crosses the BBB, mediating a normalization of the tumor vasculature, and thereby decreasing pressure and enhancing delivery of chemotherapy to the tumor.^37^ In a case series of 4 patients receiving bevacizumab and paclitaxel for breast cancer brain metastases, considerable activity was demonstrated with one complete response (CR) and 3 PR’s.^37^ Normally, the chemotherapeutic drug paclitaxel does not cross the BBB, so the addition of bevacizumab seems to have made a difference, and the preliminary results are encouraging.

Molecularly targeted agents may be important in the future prevention and treatment of brain metastases, and so far lapatinib for breast cancer and sunitinib for renal cell carcinoma seem most promising. These agents should most likely be given in combination with other treatment modalities, such as chemotherapy to obtain the optimal effect.

### Electrochemotherapy in the brain

Electrochemotherapy (ECT) is a treatment where electroporation is used to facilitate the delivery and thereby augmenting the cytotoxicity of chemotherapy. Electroporation of cells is caused by application of an electric field across the cell membrane that exceeds the cell membranes’ resting potential, leading to a destabilization of the membrane and formation of pores. Obtaining electroporation of cells requires insertion of electrodes into the tumor tissue and applying electric pulses, generating a sufficient electric field to cause pore formation. The pores in the cell membrane will be open for seconds to minutes before resealing, depending on the electrical parameters used (*e.g.* pulse duration and applied voltage), and in this time *e.g*. a chemotherapeutic drug can enter the cells easily and reach their intracellular target.^38^

**FIGURE 2.**
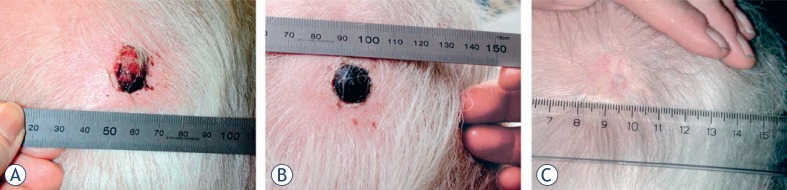
Treatment result of electrochemotherapy in the skin. One cutaneous metastasis from malignant melanoma treated with electrochemotherapy in general anaesthesia and intravenous injection of bleomycin. Pictures shows (a) Before treatment the metastases was ulcerated and caused haemorrhage, pain and discomfort, (b) 1 month after treatment the lesion is covered with a crust, needle marks in normal tissue are visible due to treatment of the tumor margin as well. Note that there is no necrosis of normal skin, and (c) 6 months after treatment the treated metastases is in CR (complete response) showing normal skin that had healed underneath the nodule. From Gehl, Ugeskrift for laeger, 2005, with permission.

The chemotherapeutic drug mostly used for ECT is bleomycin, which is highly cytotoxic once inside the cell, targeting DNA by causing double- and single strand breaks.^39^ Bleomycin is a large, hydrophilic molecule that under normal circumstances rarely enters the cells, but when combined with electroporation, the cytotoxicity is augmented at least 300 times.^40^–^44^ Bleomycin can be administered intravenously, and then the effect can be augmented in the tumor tissue by using the technique of electroporation, leading to a highly specific and localized cancer treatment. Because bleomycin is a treatment with low toxicity in single doses, this approach is readily applicable for most cancer patients.^45^ Hence, ECT as a once-only treatment for cutaneous tumors of any histology and a diameter of less than 3 cm has been shown to produce complete response rates (CR) between 73–91%.^46^–^48^ Additionally, clinical experience tells us, that the normal tissue in the tumor margins often is resistant to the effects of ECT, especially when the bleomycin is administered intravenously.^47^ In this case only a few molecules of bleomycin enter both normal and cancer cells, causing death of cancer cells, but apparently no damage to the normal cells that are still able to repair DNA damage and restore ion homeostasis after electroporation.^39^ For example, after once-only treatment of a cutaneous metastasis from malignant melanoma with ECT using intravenously administered bleomycin, the needle marks from the electrodes are visible, and it is evident that the healthy but treated tissue in the margins is unharmed, see Figure 2.^49^

Furthermore, the electric pulses cause a vascular reaction called ‘the vascular lock’, which is a local vasoconstriction possibly mediated by the sympathetic nervous system, resulting in hypoperfusion of the electroporated tissue immediately after the electric pulses, diminishing wash-out of the chemotherapeutic drug used and bleeding from the treated area.^50^,^51^

New electrode development has now made it possible to treat tumors in soft tissue inside the body, *e.g*. in the liver, gastrointestinal tract and the brain.^52^–^54^ We have shown that brain tumors in a rat model can be eliminated by ECT with this novel electrode device developed for use in the brain. The first reported preclinical study of ECT as a treatment modality for brain tumors in a rat model was published in the early 90’ties, reporting an almost double survival time in the treated rats.^55^ Acupuncture needles were then used as electrodes to treat rats inoculated with tumor cells with ECT, intravenously injected bleomycin being the chemotherapeutic drug. In our preclinical study, MRI verified tumors inoculated from glial derived tumors cells was treated with ECT, resulting in elimination of 88% of the brain tumors.^56^

**FIGURE 3.**
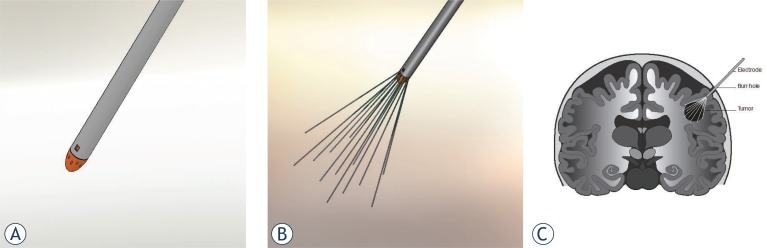
Electrochemotherapy in the human brain. Schematic drawing of the proposed electroporation procedure in the human brain

We are currently running a phase-1 trial on ECT as a palliative treatment for brain metastases using bleomycin and the novel electrode device developed especially for treatment in soft tissue (ClinicalTrials. gov, number NTC 01322100). The expandable brain electrode device developed for the phase 1 trial is seen pictured schematically in Figure 3.

In the treatment procedure, the electrode device, with the electrodes in a retracted position (Figure 3 A), will be mounted in a stereotactic frame in a specifically developed driver unit, making it possible to slowly move it forward towards the target lesion while penetrating the brain tissue. When the tip of the expandable brain electrode device reaches the correct coordinates, estimated from the MRI scan of the brain at baseline, the electrodes will be deployed into the brain metastasis (Figure 3 B, C). With the electrodes correctly placed and covering the entire tumor, the electric pulses can be delivered. After delivery of the electric pulses, the electrodes will be retracted back (Figure 3 A) and the expandable brain electrode device can be removed. Bleomycin is administered 15 minutes before delivery of the electric pulses, ensuring proper concentration of the drug in the brain tumor tissue.

ECT seems to be an attractive treatment to use in the brain, due to the known effectiveness on one side, and the limited toxic effects on normal tissue on the other. A review of the literature shows that bleomycin is quite tolerable when injected directly into the brain, most commonly causing adverse effects such as flue-like symptoms, fever, and nausea and vomiting.^57^ Common adverse effects of bleomycin administered systemically are quite similar: flu-like symptoms, fever and nausea, and mostly with high cumulative dosages, serious adverse events such as pulmonary fibrosis have been observed.^58^,^59^

As only one patient has been treated in the phase I trial so far, treatment with ECT in the brain remains experimental. However, perspectives for ECT in the brain are numerous, as it may be used as a treatment for inoperable, surgically inaccessible brain metastases, maybe in combination with conventional neurosurgery. ECT may also be used for treating the tumor bed after surgical removal of primary brain tumors such as glioblastomas, treating remaining tumor cells in the margin area responsible for local recurrence.

## Conclusions

Multiple brain metastases are increasing in incidence due to factors such as overall increasing cancer incidence, better diagnostic tools, and because improved cancer treatment causes longer survival and therefore increasing possibility of patients living long enough to develop brain metastases. Treatment options today offer limited effectiveness in patients with multiple brain metastases on the long run and neurological symptoms can be the leading problem for many of these patients. One of several new experimental treatments emerging is ECT, so far showing promising preclinical data and the first patient has been treated in an ongoing clinical trial.

## Figures and Tables

**TABLE 1.** Re-irradiation of brain metastases

**Study**	**Design (pts)**	**Radiation dose 1. treatment**	**Radiation dose 2. treatment**	**Treatment interval**	**MST**
**Shehata 1974**	Retro-spective (35 pts)	10 Gy/1 fraction or 10 Gy in 2-5 daily fractions in less than a week	Not reported	Not reported	4.7 mo
**Kurup 1980**	Retro-spective (56 pts)	18 Gy/3 fractions, 20 Gy/5 fractions or 30 Gy/10 fractions	WBRT 1 Gy/1fraction to 46 Gy/20 fractions / 5FW, at least 20 Gy in total dose	1–46 mo, Mean 6.3 mo Median 5 mo	3.1 mo
**Hazuka 1988**	Retro-spective (44 pts)	30–36 Gy/1,5-4 Gy/fraction Median 30 Gy	6–36 Gy/2–4 Gy/ fraction Median 25 Gy	Median 7.8 mo	1.7 mo
**Cooper 1990**	Retro-spective (52 pts)	30 Gy/10 fractions over 2 weeks	25 Gy/10 fractions over 2 weeks	At least 4 mo	4.9 mo
**Wong 1996**	Retro-spective (86 pts)	30 Gy/10 fractions (range 20–50.4 Gy)	WBRT 65 pt, partial brain 3 pts. Median 20 Gy/10 fractions (range 7.9–30.6 Gy)	Median 7.6 mo (range 1.5–50.6 mo)	4 mo (range 0.25–72 mo)
**Abdel-Wahab 1997**	Prospective (15 pts)	30–55 Gy Median 30 Gy	Partial brain 8.8 cm3 Median 30 Gy (range 6–30 Gy)	Median 10 mo	3.2 mo
**Sadikov 2007**	Retro-spective (72 pts)	20–30 Gy/5–10 fractions Median 20 Gy	15–25 Gy/5–12 fractions Median NR	Median 9.6 mo (range 2–37.3 mo)	4 mo (range 0–17 mo)

Abbreviations: pts-patients; WBRT- whole brain radiation therapy; MST-median survival time; mo-months (weeks converted to months for the first four studies, i.e. 4.5 weeks per month).
